# Caveolin-1 Protects Retinal Ganglion Cells against Acute Ocular Hypertension Injury via Modulating Microglial Phenotypes and Distribution and Activating AKT pathway

**DOI:** 10.1038/s41598-017-10719-x

**Published:** 2017-09-06

**Authors:** Liwei Zhang, Jiazhou Xu, Ran Liu, Wei Chen, Qishan Chen, Wenjie Hu, Lan Zhou, Ruting Zhang, Hongping Xu, Dongyue Lin, Xuri Li, Zhongshu Tang

**Affiliations:** 1 State Key Laboratory of Ophthalmology, Zhongshan Ophthalmic Center, Sun Yat-sen University, Guangzhou, 510060 China; 2 Second People’s Hospital of Yunnan Province, Kunming, Yunnan 650021 China

## Abstract

Glaucoma, a group of eye diseases, causes gradual loss of retinal ganglion cells (RGCs) and ultimately results in irreversible blindness. Studies of the underlying mechanisms of glaucoma and clinical trial are far from satisfactory. Results from a genome-wide association study have suggested that the *CAV1*/*CAV2* locus is associated with glaucoma, but this association and its potential underlying mechanisms need to be confirmed and further explored. Here, we studied the function of caveolin-1 (Cav1) in an acute ocular hypertension glaucoma model. Cav1 deficiency caused an aggregated lesion in the retina. In addition, treatment with cavtratin, a membrane permeable Cav1 scaffolding domain peptide, enhanced RGC survival. After cavtratin treatment, microglial numbers decreased significantly, and the majority of them migrated from the inner retinal layer to the outer retinal layers. Furthermore, cavtratin promoted a change in the microglia phenotype from the neurotoxic pro-inflammatory M1 to the neuroprotective anti-inflammatory M2. In a molecular mechanism experiment, we found that cavtratin activated the phosphorylation of both AKT and PTEN in cultured N9 cells. Our data highlights the neuroprotective effect of Cav1 on acute ocular hypertension and suggests that Cav1 may serve as a novel therapeutic target for the treatment of glaucoma. We further propose that cavtratin is a therapeutic candidate for glaucoma clinical trials.

## Introduction

Glaucoma is a group of eye diseases characterized by optic neuropathy and progressive degeneration of RGCs that ultimately results in irreversible blindness^1^. Previous study has estimated that approximately 80 million people worldwide will be diagnosed with glaucoma by the year 2020, making glaucoma the leading cause of irreversible blindness worldwide^2^. Elevated intraocular pressure (IOP) is currently the most widely accepted risk factor for glaucoma progression, with some other risk factors, such as age, genetic factors and vascular dysregulation^3–8^. The key pathological process of glaucoma is RGCs loss. At the molecular level, several genes, including *myocilin*, *optineurin* and *WD repeat domain*, have been associated with glaucoma^1^. In recent years, new glaucoma susceptible loci have been discovered in genome-wide association studies (GWAS). The *CAV1/2* locus was one of the first published in 2010^9^.

Cav1 and Cav2 are the structural proteins of caveolae, a flask-shaped vesicular structure located near the plasma membrane. Caveolae are found in various differentiated cells and are the most abundant in adipocytes, endothelial cells, pneumocytes, fibroblasts, smooth muscle cells and striated muscle cells^10^. In the eye, Cav1 mainly expressed in trabecular meshwork and ciliary epithelial, vascular endothelial cells, Muller cells, retinal pigment epithelial cells and photoreceptors^11, 12^. As an active intracellular vesicular structure, caveolae is associated with diverse cellular functions such as endocytosis, lipid regulation, membrane maintenance, and signal transduction^13, 14^. As the major structure protein of caveolae, Cav1 dysfunction has been linked to a wide range of diseases, such as lipodystrophy, muscular dystrophies, cardiac disease, infection, Alzheimer’s disease, Parkinson’s disease and cancer^15–17^.

The main functional domain of Cav1 is the scaffolding domain, which mediates interactions between Cav1 and many proteins, such as TrkA, EGFR, Neu/ErbB2, H-Ras, MEK, c-Src, Fyn, GPCRs, and eNOS^18–21^. A synthesized peptide called cavtratin contains the scaffolding domain and a 16-aa short internalization sequence AP, which was derived from the Drosophila transcription factor antennapedia^22^. Cavtratin works as either an analog or a dominant negative of Cav1, depending on the binding sites of the corresponding interacting proteins and their functions^23, 24^.

Like most candidates found associated with diseases in GWAS, the association, function and mechanism of *CAV1/CAV2* in glaucoma have not yet been fully explored. Some labs have studied the role of Cav1 in aqueous humor dynamics. They found that Cav-1 deficiency alters aqueous humor outflow facility and leads to increased intraocular pressure^25–27^. However, the role of Cav1 in other important aspects of glaucoma, such as RGC loss, is still largely unknown. Since Cav2 is co-expressed with Cav1, we chose Cav1 for our study^20^. Using gain- and loss-of-function approaches, we found that Cav1 protects RGCs against acute ocular hypertension stress injury. And this effect was achieved through regulating the number, phenotype and distribution of microglial cells. In the molecular mechanism study, we found the activation of AKT pathway.

## Results

### Cav1 expression is upregulated in the retina after acute ocular hypertension injury

**Figure 1:**
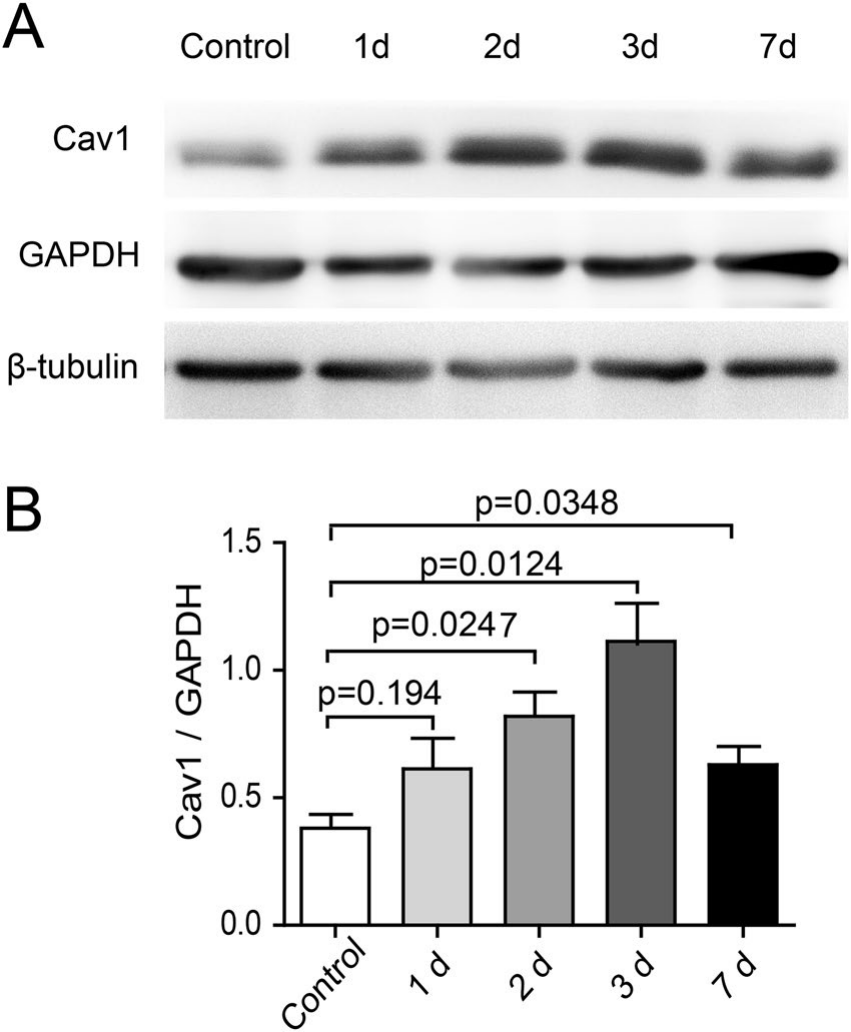
Cav1 expression is upregulated in the retina in the acute ocular hypertension injury model. IOP was elevated to 110 mm Hg, maintained for 1 hr and then allowed to return to the normal level. (**A**) Western blot of Cav1 expression at different time points during recovery. GAPDH and β-tubulin were used as the loading controls. (**B**) Statistics of the ratio of Cav1 expression level to GAPDH expression level from 3 independent experiments.

To explore whether Cav1 was involved in the pathogenesis of acute ocular hypertension injury, we examined its expression in the retina of an animal model. In mice, IOP was elevated to 110 mm Hg, maintained for 1 hr, and then allowed to return to the normal level. A daily recording showed that the IOP was in a recovering period in the first and second days after the treatment, and increased to and kept at the normal level (Supp. Figure 1). Western blot analysis showed that Cav1 expression increased gradually during the recovery period. It reached the highest level at 3d after the hypertension treatment (Fig. 1A,B). The upregulation of Cav1 expression in the retina under acute ocular hypertension suggests that Cav1 is involved in the modulation of hypertension.

### Cav1 deficiency exacerbates ocular hypertension injury

**Figure 2:**
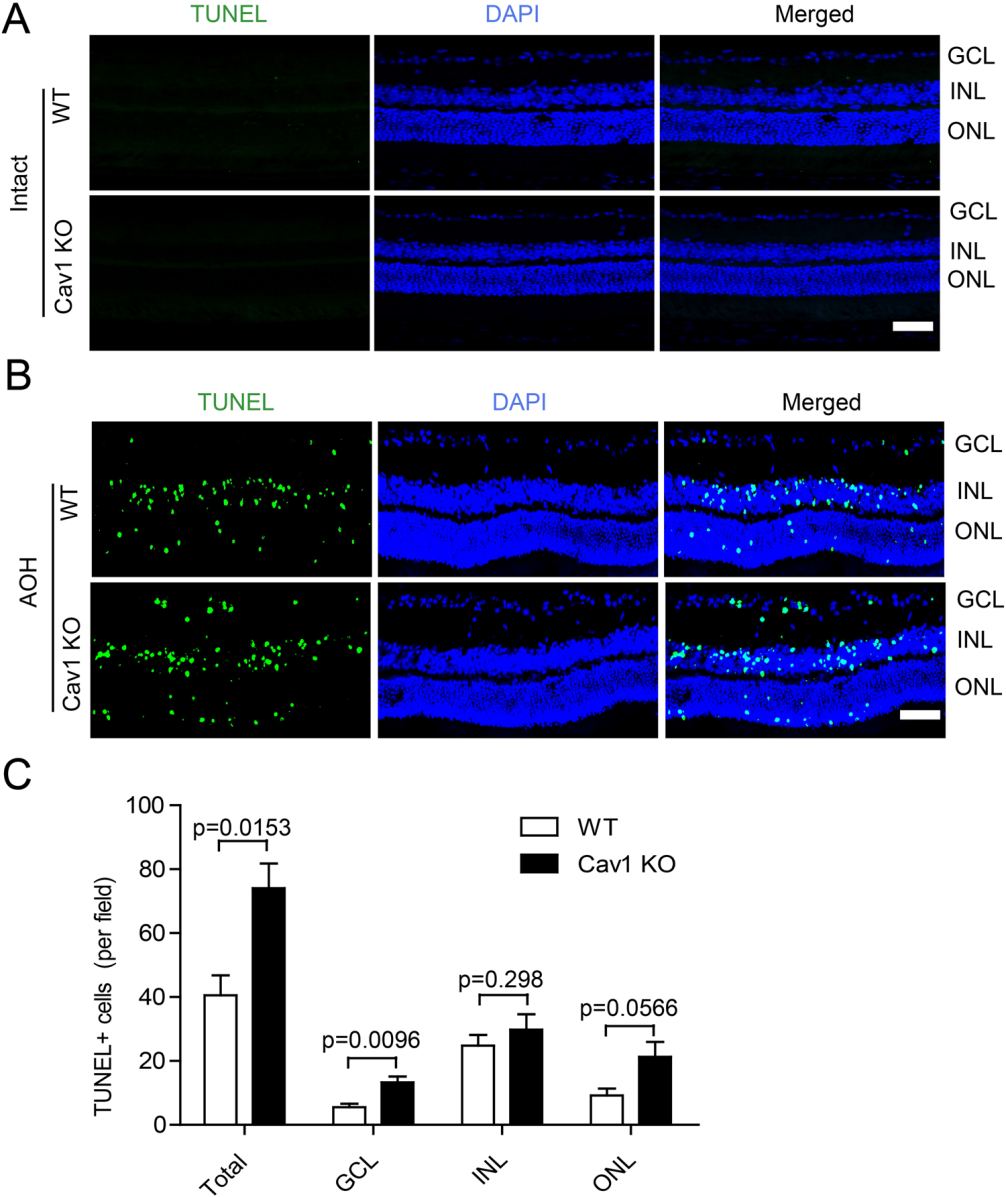
Cav1 deficiency exacerbates retinal lesions in the acute ocular hypertension. IOP was elevated to 110 mm Hg, maintained for 1 hr and then released to the normal level. Retinae were collected 3d later. (**A**) No apoptosis was detected in Cav1 KO mice under intact condition. The eyeballs of 8-wk Cav1 KO and WT mice were harvest and cryosectioned for TUNEL staining. No apoptotic cells were detected. (**B**) TUNEL staining of retinal sections from WT and Cav1 KO mice. TUNEL^+^ cells were mainly located in the laminar layers. (**C**) Statistics of the TUNEL^+^ cell numbers from 3 independent experiments. More TUNEL^+^ cells were found in the Cav1 KO mice than in the WT mice. Scale bar, 50 μm.

To explore the role of Cav1 in ocular hypertension, we performed TUNEL staining on the retinae of both WT and Cav1 KO mice after the acute ocular hypertension treatment. Under the normal condition, there was no apoptosis in the retinae of either the WT or Cavl KO adults (Fig. 2A). Under the acute ocular hypertension injury, three days after IOP recovery, the number of TUNEL^+^ cells in the Cav1 KO group was approximately 45.3% higher than that in the WT group (WT: 40.50 ± 6.273; Cav1 KO: 74.04 ± 7.775; n = 4 for both; p = 0.0153). The increase occurred in all three laminar layers (Fig. 2B,C), including the ganglion cell layer (GCL. WT group: 5.583 ± 1.006; Cav1 KO group: 13.29 ± 1.799; n = 4 for both; p = 0.0096), the inner nuclear layer (INL. WT group: 24.75 ± 3.371; Cav1 KO group 33.75 ± 7.139; n = 4 for both group; p = 0.298) and the outer nuclear layer (ONL. WT: 21.29 ± 4.657; Cav1 KO: 9.167 ± 2.189; n = 4 for both; p = 0.0566), although the increase in the INL was not statistically significant. These data indicate that loss of Cav1 exacerbates the hypertension-induced lesion in the retina and that Cav1 plays a protective role in the ocular hypertension injury model.

### Cavtratin protects RGCs against acute ocular hypertension injury

**Figure 3:**
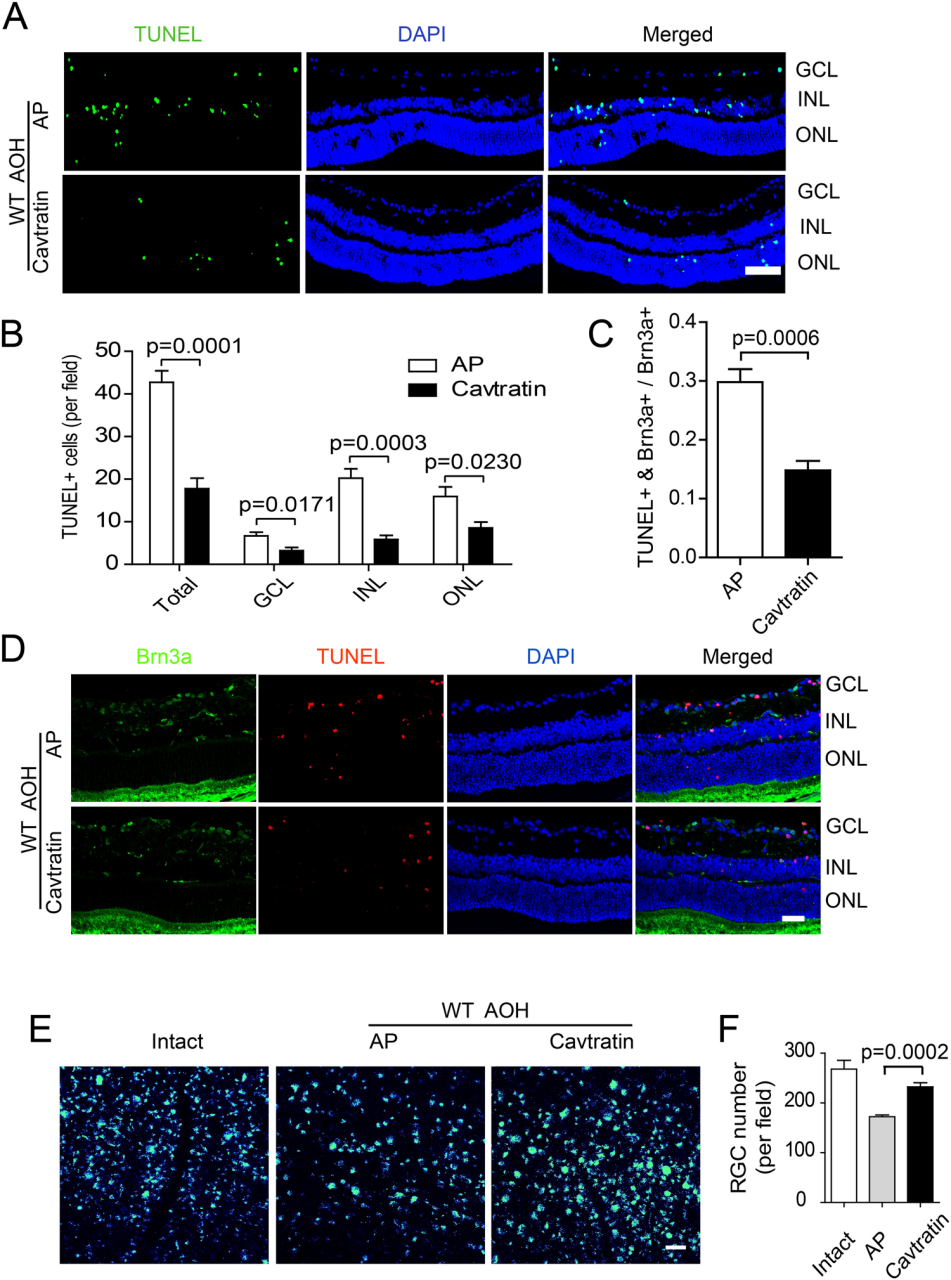
Cavtratin protects RGCs against injury in the acute ocular hypertension model. (**A,B**) Cavtratin decreased the number of apoptotic cells in the retina. AP or cavtratin was injected into the vitreous chamber 4 hr before the acute ocular hypertension treatment. Retinae were collected 3d after the hypertension. Fewer TUNEL^+^ cells were detected in the cavtratin group than in the control (AP) group in all three laminar layers. (**C,D**) RGC apoptosis decreased with application of cavtratin. RGCs were marked by immunostaining against Brn3a. TUNEL and Brn3a double labelled cells were quantified and the ratio of the double-labelled cell number to the total RGC number was used to evaluate apoptotic RGCs. (**E**,**F**) RGC survival increased in the model. RGCs were retrogradely labeled by injecting FG into the superior colliculus 7d before the acute ocular hypertension treatment. The FG labelled cells in the wholemount retinae were counted. Scale bar, 50 μm.

To confirm the protective effect suggested by the Cav1 deficient approach, we performed a gain-of-function study on the acute ocular hypertension mouse model. A 1 μl volume of 10 mM cavtratin, a peptide containing the Cav1 scaffolding domain, or the control peptide AP, was injected into the vitreous chamber 4 hr before the ocular hypertension treatment. TUNEL staining revealed that cavtratin pretreatment significantly decreased the number of apoptotic cells in all laminar layers of the retina (Fig. 3A,B. n = 5 for all. Total number: AP group: 42.73 ± 2.723; cavtratin group: 17.77 ± 2.448; p = 0.0001. GCL: AP group: 6.671 ± 0.8828; cavtratin group: 3.233 ± 0.7314; p = 0.0171. INL: AP group: 20.24 ± 2.183; cavtratin group: 5.867 ± 0.9286; p = 0.0003. ONL: AP group: 15.96 ± 2.245; cavtratin group: 8.567 ± 1.378; p = 0.0230).

To check if RGCs were indeed rescued by cavtratin treatment in the acute ocular hypertension model, we performed double staining with TUNEL and an antibody against Brn3a, an RGC marker. Double positive cells were identified in the RGC layer, which were indicative of apoptotic RGCs (Fig. 3D). Pretreatment with cavtratin decreased the number of apoptotic RGCs (Fig. 3C). The ratios of the double-labeled cells to that of the total RGCs  for each group are as follows: AP group: 0.2981 ± 0.02203; cavtratin group: 0.1483 ± 0.01588; n = 5, p = 0.0006.

To further assess the neuroprotective effect of cavtratin on RGCs in the acute ocular hypertension model, we injected Fluoro-Gold (FG) into the superior colliculus to retrogradely label RGCs, and then assessed their survival in the acute ocular hypertension injury after cavtratin pretreatment. Indeed, we found that cavtratin pretreatment increased RGC survival following the acute ocular hypertension injury (Fig. 3E,F. AP group: 172.4 ± 3.385; n = 5. Cavtratin group: 232.3 ± 8.340; n = 6. P = 0.0002). Altogether, both the loss-of-function and gain-of-function studies indicate that Cav1 plays a protective role in the retina.

### Cavtratin rescues retinae of Cav1 KO mice from the damage by acute ocular hypertension treatment

**Figure 4:**
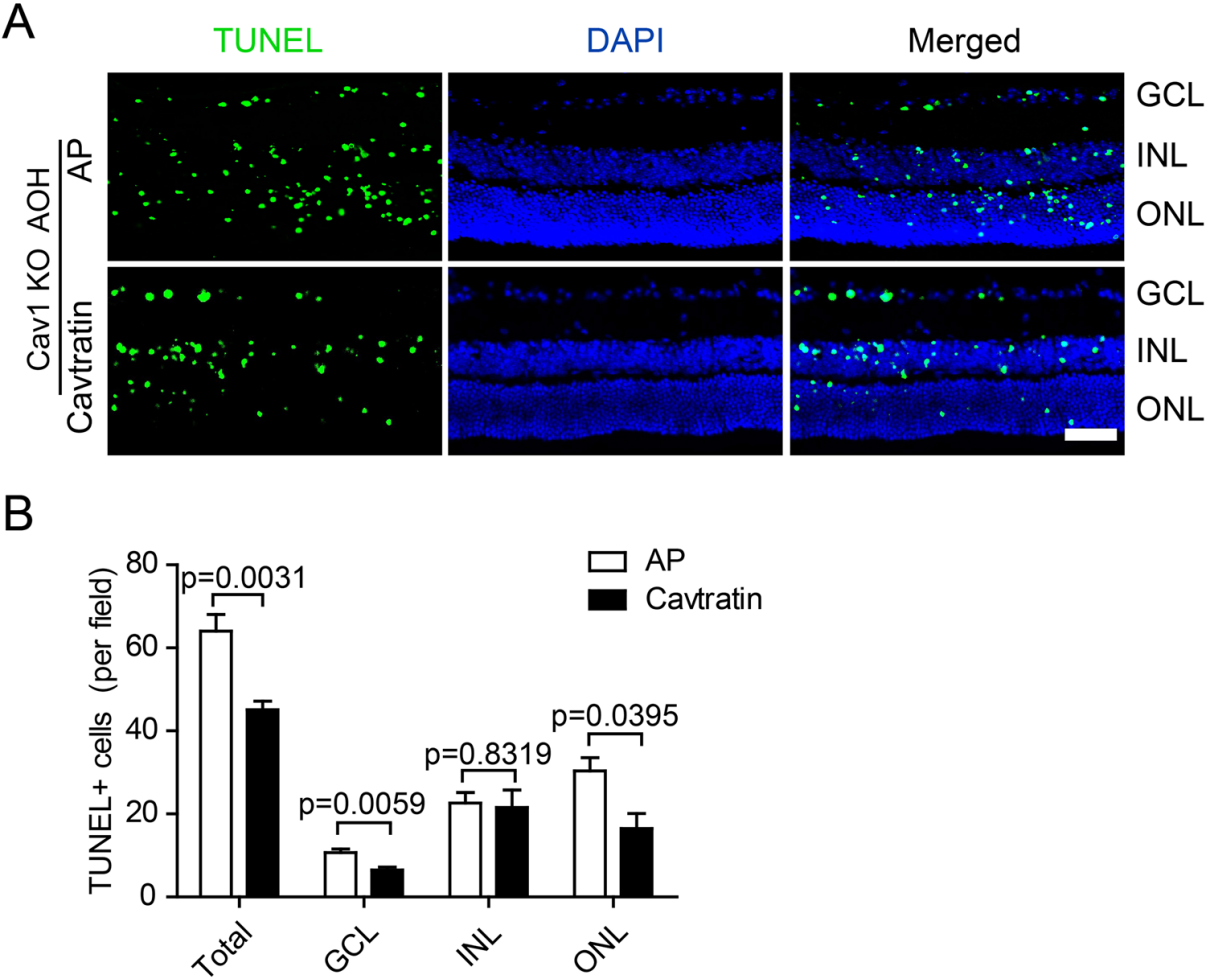
Cavtratin rescues retinae of Cav1 KO mice from the damage by acute ocular hypertension treatment. AP or Cavtratin was injected into the eye vitreous chamber 4 hr before the AOH treatment in Cav1 KO mice. The eyeballs were harvested 3d later for cryosectioning. (**A**) TUNEL staining of retinal sections. (**B**) Statistics of TUNEL^+^ cells in the retina. n = 6, Scale bar, 50 μm.

To further verify the protective role of Cav1, we applied cavtratin to the Cav1 KO mice, and checked the apoptosis in the retina after the acute ocular hypertension treatment. The number of TUNEL^+^ cells is decreased by cavtratin treatment (Fig. 4). The decrease occurred significantly in the ganglion cell layer (GCL. AP group: 10.67 ± 0.9285; Cavtratin group: 6.500 ± 0.7539; n = 6 for both; p = 0.0059) and in the outer nuclear layer (ONL. AP group: 26.10 ± 1.869; Cavtratin group: 16.52 ± 3.587; n = 6 for both; p = 0.0395). It’s interesting that, similar to the insignificant difference of apoptosis between WT and Cav1 KO in the INL under the ocular hypertension condition (Fig. 2B), the rescue effect in the INL was not different as well. The result suggests that the apoptosis in the retinae of Cav1 deficient mice can be partly rescued by cavtratin in the acute ocular hypertension model.

### Cavtratin modulates the number and distribution of microglia in the retina under acute ocular hypertension stress

**Figure 5:**
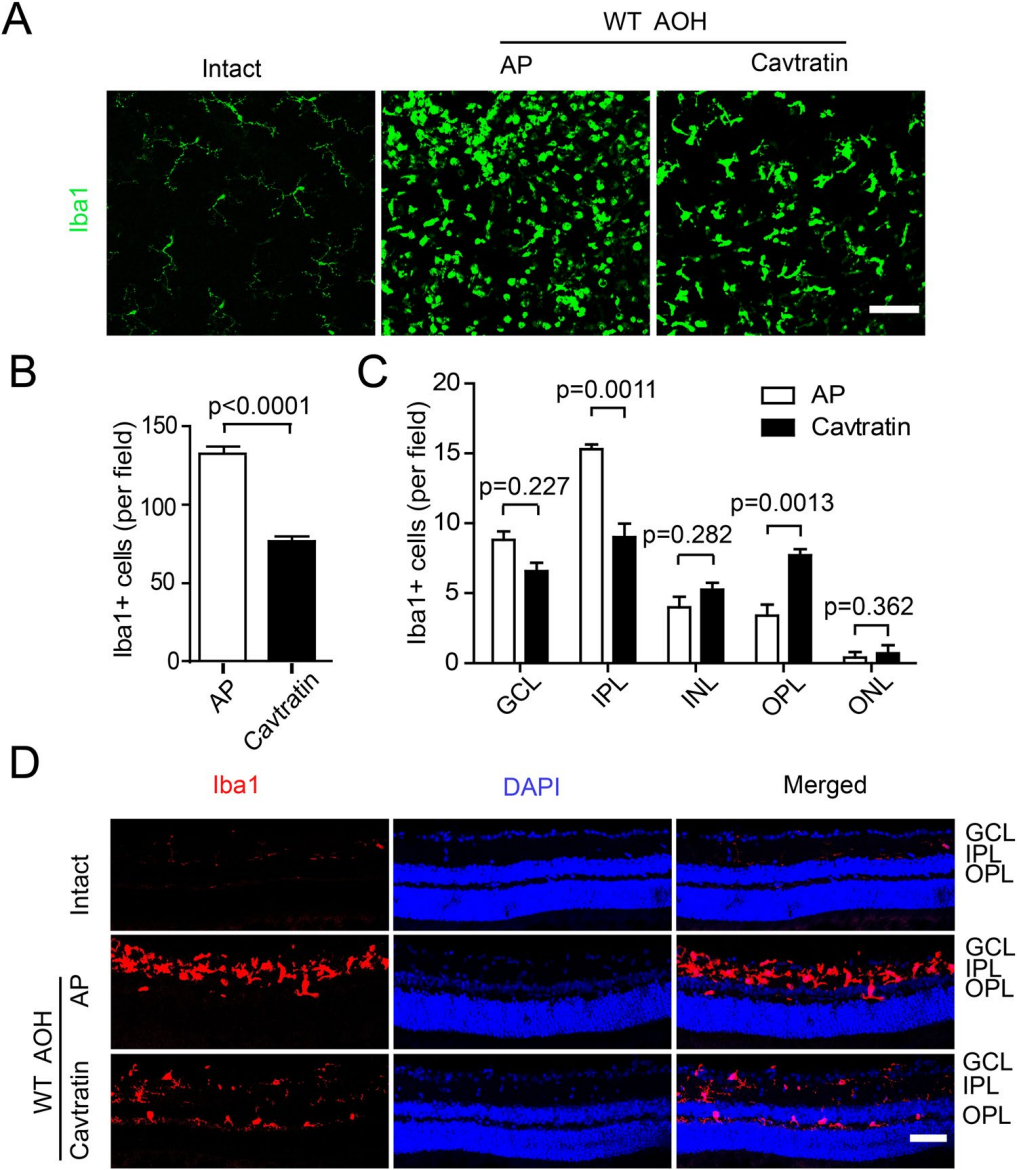
Cavtratin modulates the number and distribution of microglial cells in the retina under the acute ocular hypertension injury. Retinae were collected 3d later for analysis. (**A**) Wholemount retinae were stained with an anti-Iba1 antibody. Different density and morphology were observed in the microglial cells. (**B**) Statistics of anti-Iba1 staining in the wholemount retina. (**C**) Statistics indicate the change in microglial cell distribution across the retinal sections displayed in (**D**). (**D**) Anti-Iba1 immunostaining of retina sections. Microglial cells were mainly located in the plexiform layers. Scale bar, 50 μm.

Next, we explored the mechanism underlying the neuroprotective effect of Cav1. At the cellular level, microglial activation has been associated with many types of neuroprotective effects^28–30^. To assess the possibility that Cav1 may regulate microglia, we stained the retina with an antibody against Iba1, a microglial marker. The staining was performed on both wholemount retinae and retinal sections. The retinal wholemount staining revealed the overall microglia morphology and numbers (Fig. 5A,B). Change in morphology will be described in the next section. In the uninjured retinae, very few Iba1 positive cells were observed. In the AP group, which was pretreated with AP followed by the hypertension treatment, the number of Iba1-positive cells was markedly increased, which indicates microglial activation induced by hypertension. In the cavtratin group, which was pretreated with cavtratin followed by ocular hypertension treatment, fewer microglial cells were observed, which indicates the downregulation of the microglial population by cavtratin (AP group: 132.7 ± 5.425; cavtratin group: 76.15 ± 3.597; n = 5 for both; p < 0.0001).

The staining of the retinal sections revealed the microglial cell distribution across the retinal layers (Fig. 5C,D). In the normal retina, very few Iba1-positive cells were observed, and the majority was located in the inner plexiform layer (IPL), with fewer in the outer plexiform layer (OPL), nearly none in the GCL. In the AP group, the microglial cells were mainly located in the IPL (15.60 ± 0.5099; n = 5), with some in the GCL (8.800 ± 1.319; n = 5) and the OPL (3.000 ± 1.183; n = 5). In the cavtratin group, the microglial distribution differed from the AP group in that fewer cells were detected in the IPL (9.000 ± 0.9721; n = 6) or the GCL (6.571 ± 0.9476; n = 6), but more were found in the outer layers including the INL (5.429 ± 0.6606; n = 6), the OPL (7.714 ± 0.6061; n = 6) and the ONL (1.071 ± 0.3987; n = 6).

Altogether, these data indicate that under the treatment of cavtratin, microglial number decreases, and the majority of them migrate away from the inner retinal layers to the outer retinal layers.

### Cavtratin stimulates microglia to switch from M1 to M2 phenotypes in the retina under acute ocular hypertension injury

M1 and M2 are two phenotypes of activated microglial cells with divergent functions. Generally, in the acute hypertension model, M1 promotes death and M2 promotes survival, although activated microglial phenotypes can be more complicated^29–33^. The staining shown in Fig. 5A illustrates different microglial morphologies that can be simplified into the following 3 types: M0, M1 and M2^34, 35^. In the intact retina, the cells had small somata with thin ramified processes that resemble the quiescent M0 type. In the AP group, most cells resembled activated M1 microglia with large spheroid somata and short processes. In the cavtratin group, most cells looked similar to those in the AP group but with stretched somata, which possibly indicates the M2 phenotype. This finding suggests that the microglial cells changed phenotypes in response to cavtratin treatment.

**Figure 6:**
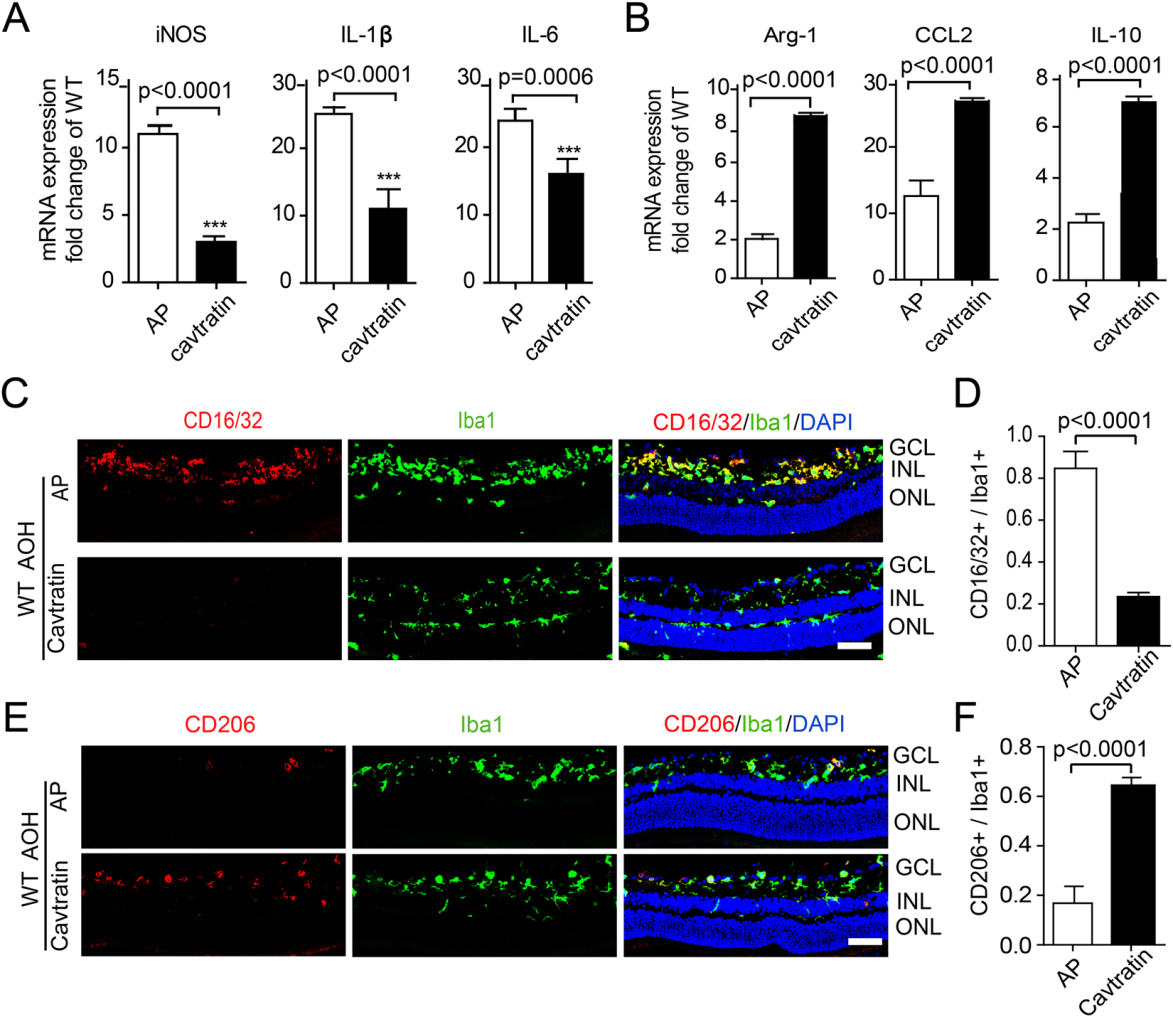
Cavtratin stimulates microglial phenotype switch from M1 to M2 in the retina under acute ocular hypertension injury. AP or cavtratin were intravitreally injected 4 hr before the establishment of acute ocular hypertension model. Retinae were collected 3d later for analysis. (**A**) Q-PCR shows the decrease in M1 marker expression in the retina. (**B**) Increase in M2 marker expression is also shown in the Q-PCR analysis. (**C,D**) Anti-CD16/32 immunostaining showed a decrease in M1 phenotype cells. (**E,F**) Anti-CD206 immunostaining shows an increase of M2 phenotype cells. Scale bar, 50 μm.

To confirm the change in the microglial phenotype, we used Q-PCR and immunostaining with M1 and M2 markers. With the cavtratin treatment, the Q-PCR results showed that the expression levels of M1 microglial markers, including iNOS, IL-1β and IL-6, were downregulated, while the expression levels of M2 markers, including Arg-1, CCL2 and IL-10, were upregulated (Fig. 6A,B). The immunostaining results revealed that the ratio of CD16/32^+^ M1 cells to the total Iba1^+^ cells decreased by approximately 4 times in the cavtratin group (Figs 6C and 6D), while the ratio of CD206^+^ cells to the total Iba1^+^ cells increased by approximately 3 times (Fig. 6E,F).

These results indicate that cavtratin modulates change in the microglial phenotype from the neurotoxic pro-inflammatory M1 phenotype to the neuroprotective anti-inflammatory M2 phenotype, which reveals the neuroprotective mechanism of cavtratin in acute ocular hypertension injury at the cellular level. It is noted that microglia activation does not happen in the Cav1 KO mice. As in the WT mice, a few Iba^+^ cells in the KO mouse retina resemble M0, but not M1 or M2 phenotype (Supp. Figure 2). It suggests that a stress condition such as the hypertension is required for the microglial activation.

### Cavtratin activates the AKT pathway

**Figure 7:**
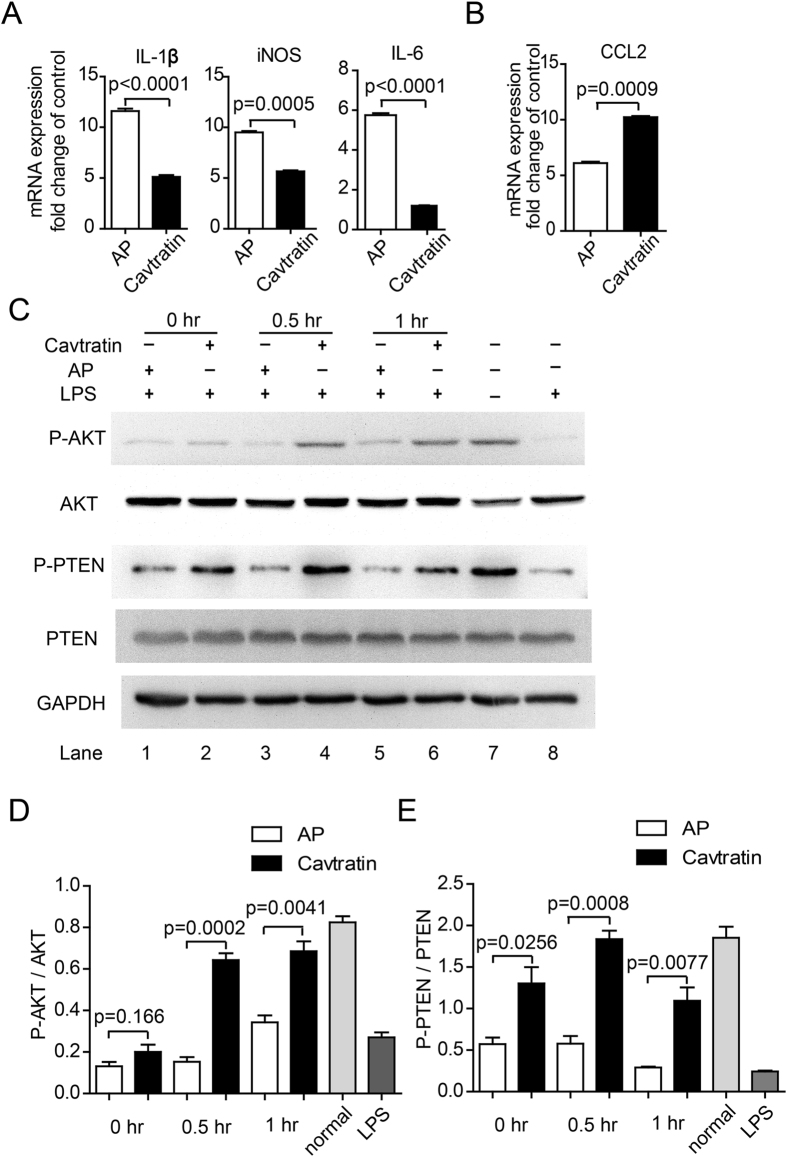
Cavtratin activates the AKT pathway in cultured N9 cells. N9 cells were pretreated with AP or cavtratin and then stimulated by LPS. (**A**) Q-PCR analysis showed the downregulation of M1 markers (iNOS, IL-1β, IL-6) by cavtratin. (**B**) Q-PCR analysis indicated the upregulation of the M2 marker CCL2 by cavtratin. (**C** and **D**) Western blot analysis indicated the increase of phosphorylation of both AKT and PTEN induced by cavtratin.

The AKT pathway is a major signaling pathway that promotes cell survival by phosphorylating a host of cellular proteins and inhibiting some death-inducing factors^36, 37^. Microglial cells have been found to be modulated by the activation of this pathway^36, 38, 39^. To determine the possibility that the AKT pathway was activated by cavtratin in microglial cells, we cultured a murine microglial cell line N9 (ATCC, US) and to see if cavtratin altered their phenotype as it did in the hypertension model. N9 cells were pretreated with 20 μM AP or cavtratin for 0.5 hr and then activated by lipopolysaccharide (LPS, 100 ng/µl) for 3 hr. The cellular effect of cavtratin on the modulation of N9 phenotype was confirmed by the downregulation of M1 markers (iNOS, IL-1β, IL-6, Fig. 7A) and the upregulation of an M2 marker CCL2 (Fig. 7B).

Western blot showed the change in AKT phosphorylation in N9 cells during LPS-induced activation. In the untreated N9 cells, AKT was highly phosphorylated (Lane 7 in Fig. 7C). After the cells were activated by LPS for 3 hr, the phosphorylation level of AKT was heavily decreased (Lane 8 in Fig. 7C). However, when the N9 cells were pretreated with cavtratin before the addition of LPS, the level of phosphorylated AKT increased (Lane 1–6 in Fig. 7C,D). This finding indicated that pretreatment with cavtratin reversed the deactivation of AKT when the N9 cells shifted to an M1 phenotype in response to LPS.

PTEN is a key negative regulator of the AKT pathway. The phosphorylation of PTEN at three specific residues (Ser380, Thr382 and Thr383) is required for its biological activity^37, 40^. Phosphorylation of PTEN decreases its activity and thus stimulates the protective activity of AKT^37^. Considering the key role of PTEN in modulating the AKT pathway in cell survival, we sought to determine whether the expression level and the phosphorylation of PTEN changed after cavtratin application to N9 cells. The result indicated that cavtratin induced the phosphorylation of PTEN while the total expression level was stable, which was in accordance with the stimulation of AKT to support the protective phenotype of microglial cells (Fig. 7C,E).

The pathway study suggests that cavtratin induces the phosphorylation of both AKT and PTEN and thus activates the AKT pathway to support the protective phenotype of microglial cells.

## Discussion

In this study, we verified the association between Cav1 and glaucoma in an acute ocular hypertension animal model. The *CAV1/2* locus on chromosome 7q31 was first reported to be associated with glaucoma in a GWAS in an Icelandic cohort and later in a Caucasian US cohort^9, 41^. However the association was not detected in an Iowa US cohort^42^. Nonetheless, the association was also detected in Japanese patients^43^. Here, we analyzed the role of Cav1 in an acute ocular hypertension model and confirmed the association of Cav1 with glaucoma in the animal model.

Regarding the role of Cav1 in glaucoma, some studies have been focused on its effect on facilitating aqueous humor outflow and thus decreasing IOP^14, 25^. Our study revealed that Cav1 protects RGCs via modulating microglial distribution and phenotype change. Here, we classified microglia with the three simplified phenotypes: quiescent M0, activated neurotoxic pro-inflammatory M1, and activated neuroprotective anti-inflammatory M2. It is important to note that microglial activation is a complicated process and their phenotypes can be quite diverse among different pathologies^31^. M1 microglial cells generally act against lesions and promote the destruction of pathogens. However, M1 microglial cells release some pro-inflammatory factors and mediators that are also neurotoxic^44, 45^. M2 microglial cells are neuroprotective and anti-inflammatory and appear after M1 for healing and repairing^45, 46^. The M1/M2 switch was observed after the application of cavtratin in our study, which reveals that cavtratin manipulated microglia to switch from neurotoxic to neuroprotective phenotypes.

Our findings also suggest the therapeutic potential of cavtratin as a treatment for glaucoma. In the clinic, glaucoma is usually treated with eye-drop drug formulations to reduce IOP, but this treatment is not always satisfactory^47^. Therefore, new drugs are desired. Glaucoma is characterized by RGC death, visual field defects and a characteristic excavated atrophy of the optic nerve^48^. Of these characteristics, RGC death is the principal effect of the disease, as the other two occur due to RGC degeneration; the optic nerve is composed of RGC axons, and the visual field is dependent on RGC function. Accordingly, RGC protection is essential for an effective glaucoma treatment. In this study, we demonstrated that RGC survival increased with the application of exogenous cavtratin. Thus, we propose cavtratin as a candidate for glaucoma treatment.

It is noted that, despite the conclusion drawn so far, there are still some limitations in this study. 1. In this paper, we focus on microglia, but other cell types are most likely to be involved, too. Immunostaining shows that the expression of Cav1 in the retina is primarily in endothelial cells and secondarily in Muller cells, which are definitely possible to be involved^11, 12^. Even some other cells with low expression level of caveolin may also play important roles^13^. Future studies will find more types of cells to establish a cellular acting loop, to identify the communicating molecules among the cells, and to define more intracellular pathways–all of the factors leading to RGC survival. 2. Instead of a scrambled sequence, we selected AP as the control of cavtratin, which may raise a concern whether it is a proper control. We think that a scrambled sequence is still a peptide with unknown but possible function. If it is not functional, more likely it will be led directly to the protein degradation pathway. Actually, Sessa Lab has tried both a scrambled sequence and the vehicle in their study on the function of cavtratin. The scrambled sequence did not give significantly different results from the vehicle^49, 50^. 3. We used the whole body Cav1 KO mice which will bring another concern about the global issues for lacking of cell specific or signaling pathway specific deletion. An alternative may be to create a *Cav1* ^*flox/flox*^ mouse line. But as most *cre-* mice are not eye-specific, the global issue cannot be solved at current technical level.

In summary, this study demonstrates that Cav1 is a neuroprotective factor in the acute ocular hypertension injury animal model. The protective effect of Cav1 was mediated by promoting the switch of the microglial phenotype from the neurotoxic pro-inflammatory M1 to the neuroprotective anti-inflammatory M2. Our result indicates that Cav1 is a novel therapeutic target in acute ocular hypertension injury and that cavtratin may be a potential drug for glaucoma clinical trials.

## Materials and Methods

### Acute ocular hypertension animal model

An acute ocular hypertension animal model was used in this study as a glaucoma model. Wild-type (C57BL/6J) mice were purchased from the Animal Laboratory of Zhongshan Ophthalmic Center. Cav1 KO mice were imported from the Jackson Laboratory (Bar Harbor, Maine), crossed on C57Bl/6 genomic background for more than 6 generations. Siblings of Cav1 KO mice and WT control mice were used for experiments. Animals were maintained on a 12-h light-dark cycle and housed in the Animal Laboratory of Zhongshan Ophthalmic Center, the Sun-yet Sen University. All animal experiments were in accordance with the guideline approved by the Institutional Animal Care and Use Committee of Zhongshan Ophthalmic Center.

To establish the acute glaucoma model, 6–8-week-old mice were anesthetized by i.p. injection of chloral hydrate. The corneas were topically anesthetized with 0.5% tetracaine hydrochloride, and the pupils were dilated with 1% tropicamide. The anterior chamber of the right eye was cannulated with a 30-gauge infusion needle connected to a normal saline reservoir, which was elevated to the height of 150 cm to maintain an intraocular pressure of 110 mm Hg for 60 min. The animals were allowed to recover for 3d before sacrifice unless otherwise specified.

### Peptides

The Cav1 scaffolding domain (DGIWKASFTTFTVTKYWFYR) was synthesized as a fusion peptide to the C-terminus of the Antennapedia 16 amino acid internalization sequence (RQIKIWFQNRRMKWKK), and referred to as cavtratin. The Antennapedia internalization sequence peptide was used as a control and referred to as AP (GL Biochem, Shanghai, China). The powder was dissolved in 1 mM acetic acid, and further diluted to 10 mM with distilled water before application.

### Tissue preparation

The eyes were enucleated, the cornea and lenses were removed, and the eyecups immersed in 4% paraformaldehyde for 30 minutes. Next, the eyecups were dehydrated in 10% sucrose, followed by 30% sucrose and embedded in OCT compounds (Tissue-Tek; Ted Pella Inc, Redding, CA, USA). The eye cups were frozen at −80 °C before cryostat sectioning.

### Retrograde labeling and quantification of RGCs

Mice were anesthetized with chloral hydrate and placed in a stereotactic apparatus. A 1 μl volume of 4% Fluoro-Gold (FG, Invitrogen) suspension was injected into the superior colliculi as previously described^51, 52^. Seven days were allotted for the retrograde transport of FG to label RGCs. After the experiment, a retinal wholemount was prepared, and FG positive RGCs were identified under a confocal fluorescence microscope (LSM710, Carl Zeiss).

### Immunostaining of wholemount retinae

The mice were euthanized, and their eyes were enucleated and fixed with freshly prepared 4% paraformaldehyde for 0.5 hr. Retinae were dissected, permeabilized in 0.5% Triton X-100 for 15 minutes, blocked in 5% BSA for 1 hr at room temperature (RT), and incubated overnight at 4 °C with a rabbit Ab against Iba-1 (ionized calcium-binding adaptor molecule 1, 1:200; Wako, Osaka, Japan), which was followed by an incubation with an Alexa Fluor labelled secondary antibody (Cell Signaling Technology, Beverly, MA) for 1 hr at RT after 3 washes with PBS. Next, the retinae were washed again and mounted on microscope slides. The retinae were examined under confocal microscopy (LSM710; Carl Zeiss). The number of Iba1 positive cell was quantified.

### Immunofluorescence for section

After air-drying for 30 minutes at RT and a brief rinse in 0.01 M PBS, the cryostat sections were permeabilized with 0.5% TritonX-100 and 5% goat serum in 0.01 M PBS for 15 min at RT. Then, the sections were incubated in the following order with primary antibodies against Iba1 (1:200; Wako), Brn3a (1:200; Millipore), CD16/32 (1:200; BD Pharmingen), or CD206 (1:200; R & D) overnight at 4 °C, Alexa Fluor-conjugated secondary antibodies for 1 hour at RT, and 0.1% DAPI for 5 minutes at RT, with washing in PBS 3 times after each incubation. Then, the sections were examined under a confocal microscope (LSM710; Carl Zeiss).

### TUNEL staining

Cryostat sections were dried and permeabilized, followed by incubation with TUNEL reaction mixture for 60 min at 37 °C (*In situ* Cell Death Detection kit; Roche Applied Science, USA). After PBS wash, the sections were incubated in 0.1% DAPI for 5 minutes followed by washing in PBS. The sections were examined under a confocal microscope (LSM710; Carl Zeiss) with the same imaging parameters. To quantify TUNEL positive cells, six images were captured for each section, and the location of each image was 300 µm from the optic nerve head. In each image, the TUNEL-positive cells were counted and averaged. Only one section was chosen from each eye, and all groups contained 4 to 6 eyes.

### Western blot

Mouse retinae or murine microglia N9 cells were washed twice with ice-cold PBS and lysed in radio immunoprecipitation assay (RIPA) buffer containing protease and phosphatase inhibitor cocktails (Thermo Scientific). Protein was quantified with a DCTM protein assay kit (Bio-Rad), loaded on an 8–12% SDS-PAGE gel and transferred to a PVDF membrane (BIO-RAD). After blocking in 5% BSA, the membrane was incubated with a primary antibody against Cav1 (1:1000; Cell Signaling Technology (CST), Beverly, MA), Phospho-PTEN (Ser380/Thr382/383; 1:1000; CST), PTEN (1:1000; CST), AKT (pan; 1:1000; CST), or Phospho-AKT (Ser473; 1:1000; CST) at 4 °C overnight followed by an incubation with an HRP-conjugated secondary antibody (1:5000) for 1 hr at RT, with 3 TBS wash after each incubation. GAPDH (Sigma) served as the loading control. Super Signal Pico (Thermo Scientific) or Immobilon Western Chemiluminescent HRP substrate (Merck Millipore) was used and the PVDF membranes were visualized using a G-BOX (Syngene) image capture system. All experiments were independently performed for 3–4 times. For the Western blot, the band intensity was determined with ImageJ (NIH, Bethesda, MD).

### RNA isolation and real-time PCR

Total RNA from either mouse retinae or N9 cells was isolated using TRIzol according to the manufacturer’s instruction (Life Technology). A total of 1000 ng RNA was reverse transcribed using the PrimeScript RT reagent Kit (TAKARA Biotechnology). Real-time PCR (QuantStudio 6 Flex, Life technologies) was performed using a SYBR® Green Master Mix (Roche). Gene expression levels were normalized to actin mRNA levels and the data were calculated using the 2^−ΔΔCT^ method. All experiments were performed in triplicate and repeated twice. The qPCR procedures comprised 50 °C for 2 min and 95 °C for 4 min during the hold stage, followed by 95 °C for 15 sec and 60 °C for 60 sec for 50 cycles. Real-time PCR primers are listed in Table 1.

**Table 1:** List of primers for Q-PCR.

Gene	Forward (5′-3′)	Reverse (5′-3′)
iNOS	CAAGCACCTTGGAAGAGGAG	AAGGCCAAACACAGCATACC
Il-1β	TGAAATGCCACCTTTTGACAG	CCACAGCCACAATGAGTGATAC
Il-6	TGGAGTCACAGAAGGAGTGGCTAAG	TCTGACCACAGTGAGGAATGTCCAC
Arg-1	CTCCAAGCCAAAGTCCTTAGAG	AGGAGCTGTCATTAGGGACATC
Ccl-2	TACAAGAGGATCACCAGCAGC	ATTCCTTCTTGGGGTCAGCAC
Il-10	CTTACTGACTGGCATGAGGATCA	GCAGCTCTAGGAGCATGTGG
β-Actin	GGCACCAGGGCGTGATGG	GTCTCAAACATGATCTGGGTC

### Data analysis

Quantification of the above mentioned staining has been described in the corresponding methods. Statistical analysis was performed with unpaired two-tailed Student’s t-test using Graph Pad Prism software (Graph Pad Prism 5, San Diego, California). Data were expressed as the mean ± SEM. P < 0.05 was considered the threshold for statistical significance.

### Nonstandard abbreviations used

Cav1, caveolin-1; DAPI, 4′,6-diamidino-2-phenylindole; FG, Fluoro-Gold; GCL, ganglion cell layer; Iba-1, ionized calcium-binding adaptor molecule 1; INL, inner nuclear layer; IOP, intraocular pressure; IPL, inner plexiform layer; LPS, lipopolysaccharide; ONL, outer nuclear layer; OPL, outer plexiform layer; PI3K, phosphatidylinositol 3-kinase; PIP-3, phosphoinisitidylinositol-3,4,5-triphosphate; PTEN, the phosphatase and tensin homolog deleted on chromosome 10; RGC, retinal ganglion cell; RT, room temperature.

## Electronic supplementary material

supplementary figures
