# Supplementary material for: Caveolin-1 Protects Retinal Ganglion Cells against Acute Ocular Hypertension Injury via Modulating Microglial Phenotypes and Distribution and Activating AKT pathway
Source: Sci Rep. 2017 Sep 6;7:10716. doi: 10.1038/s41598-017-10719-x (PMC5587691; doi:10.1038/s41598-017-10719-x)
Supplement: Supplementary file 1 — supplementary figures [file 41598_2017_10719_MOESM1_ESM.pdf]

# Caveolin-1 Protects Retinal Ganglion Cells against Acute Ocular Hypertension Injury via Modulating Microglial Phenotypes and Distribution and Activating AKT pathway

Liwei Zhang[1, 2], Jiazhou Xu[1], Ran Liu[1], Wei Chen[1], Qishan Chen[1], Wenjie Hu[1], Lan Zhou[1], Ruting Zhang[1], Hongping Xu[1], Dongyue Lin[1], Xuri Li[1], Zhongshu Tang[1,*]

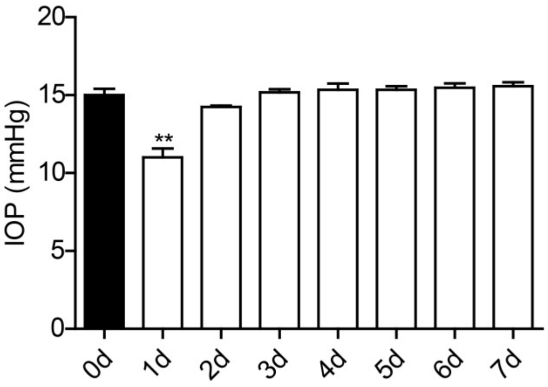


Supp. Fig. 1. IOP kept at the normal level after the hypertension treatment. IOP were measured daily before and after the establishment of acute ocular hypertension model. The IOP reduced significantly right after the treatment because of the loss of aqueous humor. However, it recovered to the normal level thereafter. n=4, p=0.0013 for 0d vs 1d.

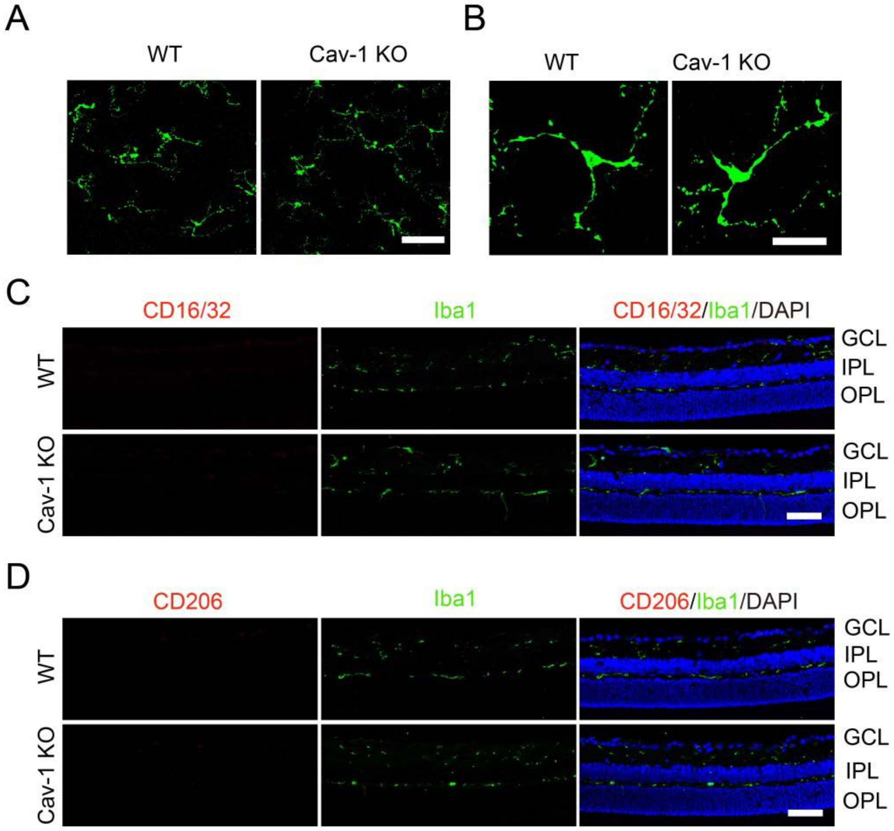


Supp. Fig. 2. Cav1 KO mouse retina does not show microglia activation under intact condition. (A, B) 8-wk mouse wholemount retinae were preceded for immunostaining against Iba1. (C). Retinal sections stained with M1 marker CD16/32. (D). Retinal sections stained with M1 marker CD206. Scale bar: 50 μm in A, C and D; 20 μm in B.
